# Supplementary material for: State-trait interactions in regulatory focus determine impulse buying behavior
Source: PLoS One. 2021 Jul 2;16(7):e0253634. doi: 10.1371/journal.pone.0253634 (PMC8253419; doi:10.1371/journal.pone.0253634)
Supplement: S1 File — (PDF) [file pone.0253634.s001.pdf]

Additional Analyses

The data presented in the main body of the text is relevant to additional questions beyond those discussed in the main text. Specifically, two prior findings may be addressed: One, that time pressure decreases unplanned buying (e.g. Iyer 1989), and two, that trait buying impulsiveness has a stronger impact on impulsive buying behavior when cognitive resources are lacking (Vohs & Faber, 2007). In addition, the main analyses presented in the text can be applied separately to healthy and unhealthy products.

Replication Analyses: Time Pressure and Cognitive Resources

The first finding has been demonstrated in several studies to date (Beatty & Ferrell, 1998; Iyer, 1989). The general explanation given for this effect is that being under time pressure forces individuals to adhere to their purchasing script in a retail environment and thereby prevents browsing opportunities. In the current experiments, there is no possible influence of browsing per se (in the sense of finding products that one otherwise would not have found), nor does any participant have a purchasing script. Therefore, the experiments are not directly comparable. However, it might also be argued that time pressure leads to a general tendency to reject impulse buying opportunities or alternatively, that time pressure can increase impulse buying under certain conditions, such as when the implicit attitudes towards the products are positive (Friese et al., 2008). Data from Experiment 1 speaks to these possibilities. Participants' impulse spending did not differ as a function of time pressure ($t(106) = .51$, $p = .614$, $d = .10$). This does not change even if the entire sample over both experiments is included in the analysis with budget percentage spent as the criterion ($t(248) = .64$, $p = .525$, $d = .08$). Therefore, these data do not support a direct effect of the availability of cognitive resources on impulse buying.

The second finding (that trait buying impulsiveness has a stronger impact on impulsive buying behavior when cognitive resources are lacking) was shown in two ego-depletion experiments (Vohs & Faber, 2007). The authors argued that lack of available self-control resources would lead to more impulse spending. They attributed their failure to find an effect of trait buying impulsiveness in their no depletion condition to participants' intact ability to regulate their spending. As self-control resources have been linked to working memory resources and reflective resources (Hofmann et al., 2011; Vohs, 2006), an analogous effect would be expected in experiment 2. A regression model that included mean-centered BIS score, cognitive load condition as a dummy variable (0 = low load, 1 = high load) and their interaction term with amount of money spent as the criterion did not achieve significance ($F(3,138) = .81$, $p = .491$). Neither the predicted interaction effect ($B = -.004$, $SE = .214$, $p = .985$) nor the main effect of the BIS score ($B = .068$, $SE = .151$, $p = .651$) achieved significance. A similar model over the entire sample of both experiments with percentage of money spent as a criterion also failed to achieve significance ($F(3,246) = .98$, $p = .402$; interaction: $B = -.027$, $SE = .037$, $p = .462$; BIS main effect: $B = -.039$, $SE = .025$, $p = .127$). These data do not support a greater effect of trait buying impulsiveness on impulse spending under circumstances where resources to self-regulate are impaired, insofar as one accepts the assumption that cognitive load and/or time pressure are functionally identical to ego depletion with regard to limiting self-control resources.

Exploratory Analyses: Differentiating By Product Type

Finally, as noted by an anonymous reviewer, our selection of products for the store allowed us to differentiate between relatively healthy and unhealthy products. Specifically, it can be argued that our categories of jelly babies, chocolate bars, potato chips, and sweetened soft drinks would likely be considered unhealthy, whereas the categories of fruit smoothies, drinking yoghurts, fruit

and nut trail mixes, and water/carbonated juices would likely be considered relatively healthy. Beyond the conflict of positive and negative outcomes inherent in impulse buying situations (i.e. the pleasure of consuming the product vs. the pain of losing the money), unhealthy foods in particular are likely to lead to increased conflicts due to their additional attributes (being perceived as particularly tasty vs. having detrimental health effects; e.g. Gillebaart et al., 2016). Thus, it could be argued that regulatory focus effects in general should be increased for unhealthy foods compared to healthy foods, as both potential positive outcomes (pertaining to a promotion focus) and negative outcomes (pertaining to a prevention focus) of consumption are greater in magnitude. In order to investigate this possibility, the regression models calculated to investigate state-trait interaction in regulatory focus were recalculated separately for percentage of budget spent on healthy products vs. unhealthy products. As these analyses were conducted post-hoc, the risk of alpha inflation was higher; thus, only the entire dataset of both experiments was analyzed to ensure sufficient statistical power. The results are presented in Tables SPL1 and SPL2.

For healthy products, the promotion focus model shows no significant effects, although the promotion fit interaction term is descriptively negative, in line with the results from the combined analysis (see Figure SPL1). However, the prevention focus model shows a significant fit effect: individuals with a high chronic prevention focus spend a significantly greater percentage of their compensation on healthy products in a situational promotion focus (B = -.106, SE = .042, p = .013), whereas individuals with a low chronic prevention focus do not (B = .057, SE = .043, p = .188). The three-way interaction with resource constraint observed in the combined interaction does not manifest (see Figure SPL2).

For the unhealthy products, there is a significant promotion fit effect such that under a high chronic promotion focus, a situational promotion focus increases spending (B = -.092, SE = .035,

p = .010), whereas it does not under a low chronic promotion focus (*B* = .015, *SE* = .036, *p* = .675, see Figure SPL1). In the prevention model, the three-way interaction term achieves significance. The results are similar to those of the combined analysis: only when participants are unconstrained and have a high chronic prevention focus does the effect of situational regulatory focus manifest (*B* = -.081, *SE* = .039, *p* = .040); if participants are constrained or have a low chronic prevention focus, there is no such effect (all |*Bs*| < .042, all *ps* > .191, see Figure SPL3).

Table SPL1

Unstandardized regression coefficients and statistics for exploratory regression models. Criterion: percentage of budget spent on healthy products.

	B	*SE*	*t*	*p*
Promotion				
Buying Impulsiveness	.004	.014	.29	.771
Chronic Prevention	-.007	.021	-.31	.754
Resource Constraint	.020	.030	.65	.516
RF Condition	-.026	.031	-.83	.408
Chronic Promotion	.039	.042	.93	.355
RC*RF	-.035	.044	-.79	.428
Chronic Promotion*RC	.046	.068	.68	.497
Chronic Promotion*RF	-.056	.060	-.93	.354
Chronic Promotion*RC*RF	-.040	.090	-.44	.657
Prevention				
Buying Impulsiveness	.001	.013	.11	.913
Chronic Promotion	.016	.022	.70	.484
Resource Constraint	.016	.030	.52	.602
RF Condition	-.024	.031	-.78	.434
Chronic Prevention	.054	.037	1.45	.148
RC*RF	-.036	.043	-.83	.409
Chronic Prevention*RC	-.022	.054	-.41	.680
Chronic Prevention*RF	-.163	.059	-2.76	.006
Chronic Prevention*RC*RF	.088	.083	1.07	.286

Table SPL2

Unstandardized regression coefficients and statistics for exploratory regression models. Criterion: percentage of budget spent on unhealthy products.

	B	*SE*	*t*	*p*
Promotion				
Buying Impulsiveness	.021	.011	1.84	.067
Chronic Prevention	.082	.035	2.38	.018
Resource Constraint	-.046	.025	-1.83	.068
RF Condition	-.038	.026	-1.49	.138
Chronic Promotion	.025	.017	1.46	.146
RC*RF	.055	.036	1.53	.127
Chronic Promotion*RC	-.080	.056	-1.43	.154
Chronic Promotion*RF	-.107	.050	-2.16	.032
Chronic Promotion*RC*RF	.093	.074	1.26	.211
Prevention				
Buying Impulsiveness	.017	.011	1.50	.135
Chronic Promotion	.014	.018	.75	.452
Resource Constraint	-.048	.025	-.190	.058
RF Condition	-.037	.026	-1.45	.150
Chronic Prevention	.062	.031	2.01	.046
RC*RF	.054	.036	1.49	.137
Chronic Prevention*RC	-.054	.045	-1.19	.234
Chronic Prevention*RF	-.096	.49	-1.96	.051
Chronic Prevention*RC*RF	.149	.069	2.16	.032

These results appear to broadly confirm the ad-hoc prediction that regulatory focus effects would be stronger for unhealthy products. Healthy products only showed a trend towards a promotion fit effect, but this trend achieved significance for unhealthy products. With regard to prevention fit, the results are more complex: a fit effect manifested for the healthy products, but the same fit effect occurred for unhealthy products only when participants were cognitively unconstrained. Thus, it appears that the results found in the combined analysis are driven more by the unhealthy products, although the pattern is descriptively similar in the healthy products. It is

Figure SPL1

Regression estimates for promotion focus models (healthy/unhealthy products)

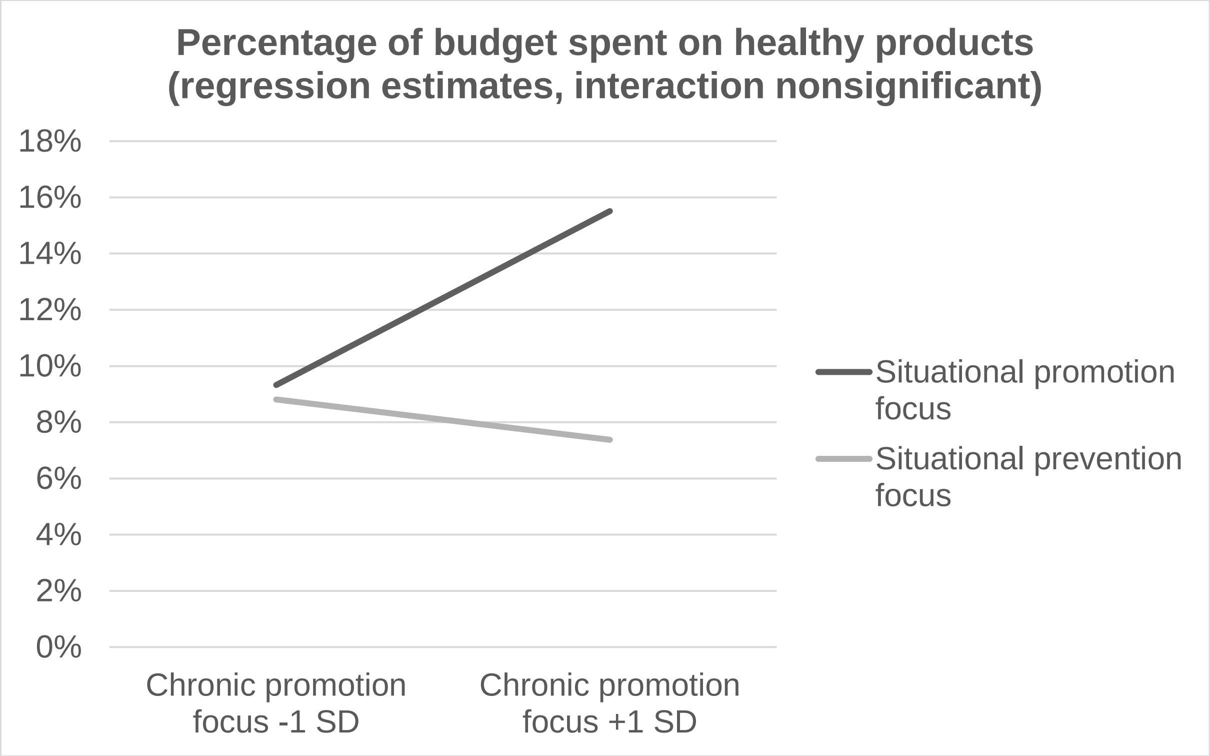

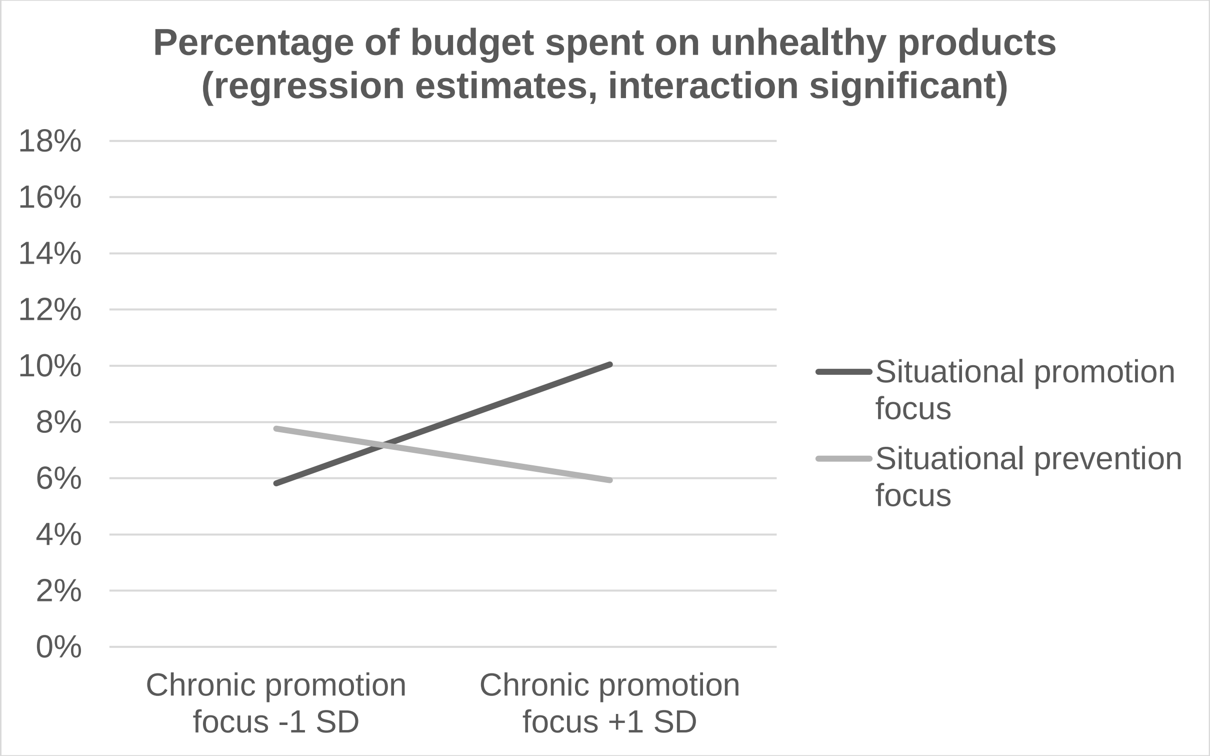

Figure SPL2

Regression estimates for prevention focus model (healthy products)

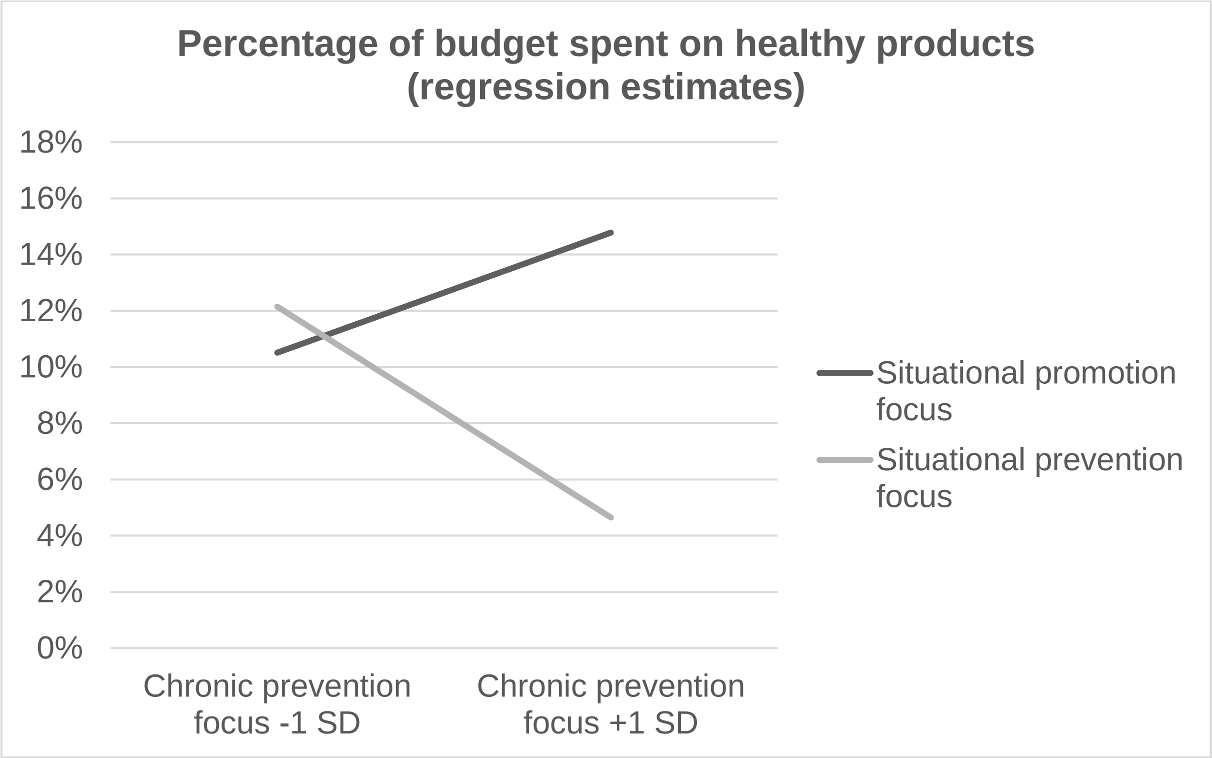

important to note, however, that these conclusions are derived from post-hoc tests and that some findings would be rendered nonsignificant by appropriate alpha correction.

On a theoretical level, bearing in mind the relative fragility of the effects, these findings tentatively suggest that promotion goals associated with impulsive snack buying are primarily concerned with immediate hedonic gratification from consumption (cf. Sengupta & Zhou, 2007), in line with stronger findings for the (presumably more immediately gratifying) unhealthy products. On the other hand, prevention goals in such a context appear to be more complex. Chronically prevention-focused individuals are likely to spontaneously notice and be motivated to consider the risks of any decision, but situational regulatory focus may override such spontaneous inclinations (Sengupta & Zhou, 2007). For healthy products, the main risk in impulsively buying is unnecessarily expending money, but for unhealthy products, there is an additional risk to one's

Figure SPL3

Regression estimates for prevention focus model split by constraint (unhealthy products)

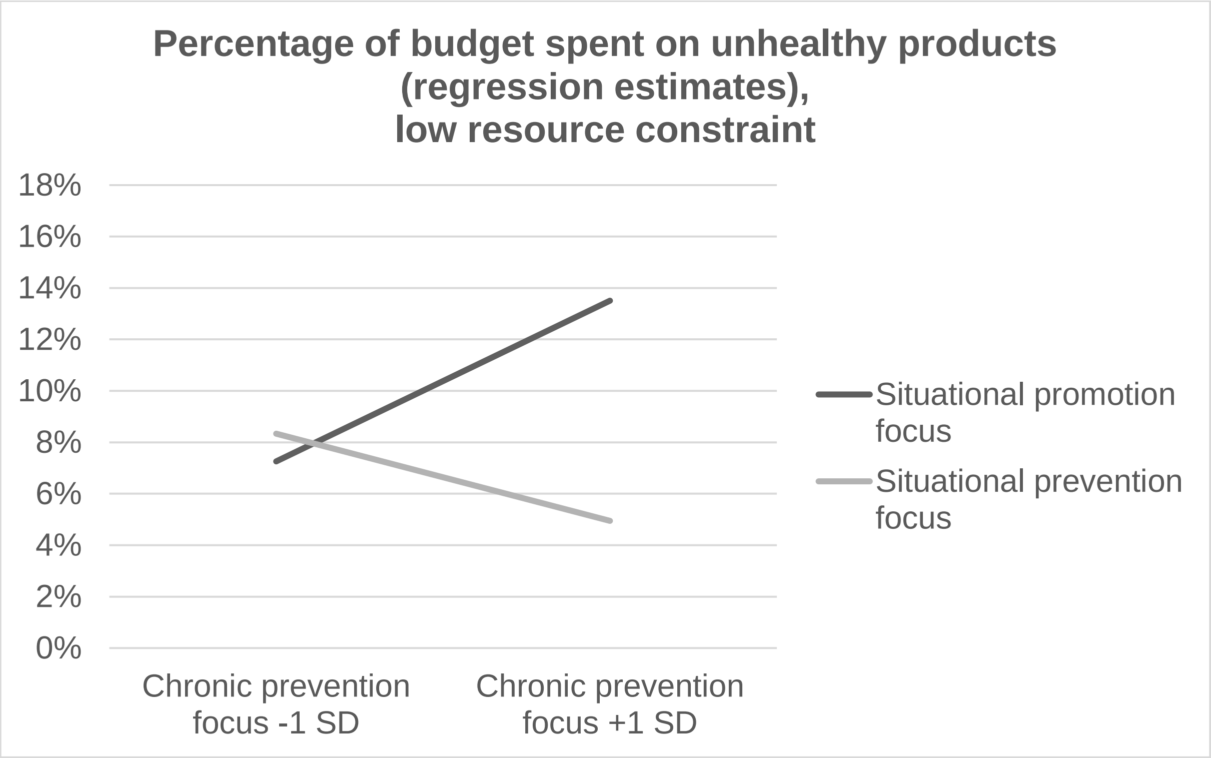

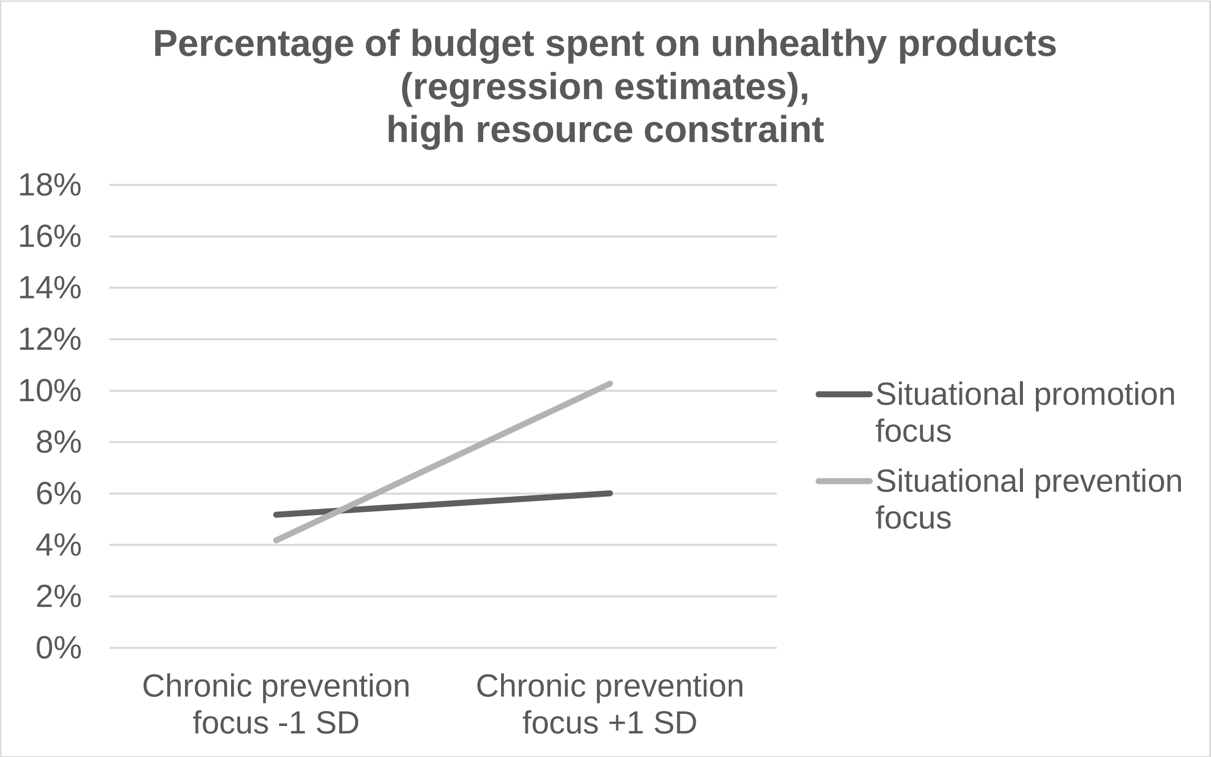

well-being. Thus, the situational promotion focus induced in the experiments might not have been strong enough to override both of these risks without additional elaborative processing.

As these results are relatively weak from a statistical standpoint and the theoretical explanation makes several notable assumptions, it would be inappropriate to draw strong inferences from these data. However, these exploratory analyses suggest several hypotheses for future research. In particular, previous findings that a situational prevention focus manipulation may override a spontaneous promotion focus (Sengupta & Zhou, 2007) might be extended to the converse case (a situationally induced promotion focus overriding a spontaneous prevention focus). In addition, it bears investigation whether multiple activation cues for spontaneous regulatory focus require additional cognitive resources to be overridden by a situational regulatory focus induction.

References

Beatty, S. E., & Ferrell, M. E. (1998). Impulse buying: Modeling its precursors. *Journal of Retailing*, *74*(2), 169–191.

Friese, M., Hofmann, W., & Wänke, M. (2008). When impulses take over: Moderated predictive validity of explicit and implicit attitude measures in predicting food choice and consumption behaviour. *British Journal of Social Psychology*, *47*(3), 397–419. https://doi.org/10.1348/014466607X241540

Gillebaart, M., Schneider, I. K., & Ridder, D. T. D. D. (2016). Effects of Trait Self-Control on Response Conflict About Healthy and Unhealthy Food. *Journal of Personality*, *84*(6), 789–798. https://doi.org/10.1111/jopy.12219

Hofmann, W., Friese, M., Schmeichel, B. J., & Baddeley, A. D. (2011). Working memory and self-regulation. In *Handbook of self-regulation: Research, theory, and applications, 2nd ed* (pp. 204–225). Guilford Press.

Iyer, E. S. (1989). Unplanned purchasing: Knowledge of shopping environment and time pressure. *Journal of Retailing*, *65*(1), 40-. Academic OneFile.

Sengupta, J., & Zhou, R. (2007). Understanding Impulsive Eaters' Choice Behaviors: The Motivational Influences of Regulatory Focus. *Journal of Marketing Research*, *44*(2), 297–308. https://doi.org/10.1509/jmkr.44.2.297

Vohs, K. D. (2006). Self-Regulatory Resources Power the Reflective System: Evidence From Five Domains. *Journal of Consumer Psychology*, *16*(3), 217–223. https://doi.org/10.1207/s15327663jcp1603_3

Vohs, K. D., & Faber, R. J. (2007). Spent Resources: Self-Regulatory Resource Availability Affects Impulse Buying. *Journal of Consumer Research*, *33*(4), 537–547. https://doi.org/10.1086/510228
