# Supplementary material for: State-trait interactions in regulatory focus determine impulse buying behavior
Source: PLoS One. 2021 Jul 2;16(7):e0253634. doi: 10.1371/journal.pone.0253634 (PMC8253419; doi:10.1371/journal.pone.0253634)
Supplement: S2 File — (PDF) [file pone.0253634.s002.pdf]

Products and Prices for the Impulse Buying Task

Product category	Price	Individual product selection
Soft drinks	0.75€	0.5l bottle Coca-Cola<br>0.5l bottle Mezzo Mix<br>0.5l bottle orange Fanta
Potato chip style snacks	0.80€	Packet Alnatura pretzels<br>Packet Funnyfrisch paprika potato chips
Gummi bear style snacks	0.65€	Packet Haribo “Goldbären”<br>Packet Katjes “Yoghurt-Gums”
Yoghurt drinks	0.70€	Alnatura Mango drinking yoghurt<br>Müller Pineapple-coconut buttermilk<br>Alnatura raspberry Lassi
Nut or fruit mix	0.95€	Packet nut/raisin mix<br>Packet dried banana chips<br>Packet dried cranberries
Chocolate bars	0.65€	2x mini Snickers bar<br>Twix Xtra bar<br>2x mini Mars bar
Fruit smoothies	0.60€	Cherry-banana smoothie<br>Mango-orange smoothie
Water and light drinks	0.35€	0.75l bottle light apple-grape-cranberry soda<br>1l bottle carbonated water<br>0.75l bottle lemon-flavored carbonated water
