# Supplementary material for: State-trait interactions in regulatory focus determine impulse buying behavior
Source: PLoS One. 2021 Jul 2;16(7):e0253634. doi: 10.1371/journal.pone.0253634 (PMC8253419; doi:10.1371/journal.pone.0253634)
Supplement: S3 File — (PDF) [file pone.0253634.s003.pdf]

Methodological Data Appendix

Table MDA1

Intercorrelations and descriptive data for regression predictors across experiments.

	M (*SD*) Experiment 1	*M* (*SD*) Experiment 2	*M* (*SD*) Mega-analysis
Chronic promotion focus	3.64 (.47)	3.53 (.52)	3.58 (.50)
Chronic prevention focus	3.86 (.46)	3.76 (.57)	3.80 (.53)
Buying impulsiveness	2.52 (.77)	2.42 (.89)	2.46 (.84)

Table MDA2

Descriptive data for money spent in € (relevant to ANCOVA in Experiment 1).

	Promotion focus (*n*=56) *M (SD)*	Prevention focus (*n*=52) *M (SD)*
Low Time Pressure (*n*=53)	1.70 (2.05) *n*=28	.50 (1.08) *n*=25
High Time Pressure (*n*=55)	1.74 (2.12) *n*=28	.87 (1.70) *n*=27

In Experiment 1, the covariate BIS in the ANCOVA did not achieve significance ($F(1,103) = .89$, $p = .248$, $\eta_p^2 = .009$). Repeating the analysis without the covariate did not substantively change the results (main effect situational regulatory focus: $F(1,104) = 8.85$, $p = .004$, $\eta_p^2 = .078$, no other effects significant, all *Fs* < 1, all *ps* > .55, all $\eta_p^2 < .005$).

Table MDA3

Descriptive data for money spent in € (relevant to ANCOVA in Experiment 2).

	Promotion focus (*n*=72)<br>*M (SD)*	Prevention focus (*n*=70)<br>*M (SD)*
Low Cognitive Load (*n*=70)	.99 (1.14)<br>*n*=39	1.07 (1.34)<br>*n*=31
High Cognitive Load (*n*=72)	.69 (.98)<br>*n*=33	.81 (1.01)<br>*n*=39

In Experiment 2, the covariate BIS in the ANCOVA did not achieve significance ($F(1,137) = .08$, $p = .775$, $\eta_p^2 = .001$). Repeating the analysis without the covariate did not substantively change the results (no effects significant, all *Fs* < 2.2, all *ps* > .14, all $\eta_p^2 < .016$).

Table MDA4

Descriptive data for ratio of money spent (relevant to ANCOVA in Mega-Analysis).

	Promotion focus (*n*=128)<br>*M (SD)*	Prevention focus (*n*=122)<br>*M (SD)*
Low Resource Constraint (*n*=123)	.216 (.256)<br>*n*=67	.150 (.239)<br>*n*=56
High Resource Constraint (*n*=127)	.189 (.255)<br>*n*=61	.146 (.219)<br>*n*=66

In the Mega-Analysis, the covariate BIS in the ANCOVA did not achieve significance ($F(1,245) = 1.62$, $p = .204$, $\eta_p^2 = .007$). Repeating the analysis without the covariate did not substantively change the results (main effect situational regulatory focus: $F(1,246) = 3.13$, $p = .078$, $\eta_p^2 = .013$, no other effects significant, all *Fs* < 1, all *ps* > .60, all $\eta_p^2 < .002$)

Table MDA5

Unstandardized regression coefficients and statistics for models calculated in Experiment 1.

	B	*SE*	*t*	*p*
Model for H2				
Buying Impulsiveness	.258	.240	1.07	.287
Time Pressure	.181	.357	.51	.613
Chronic Prevention	-.092	.535	-.17	.864
Chronic Promotion	.757	.529	1.43	.156
Chronic Prevention*TP	-.625	.794	-.79	.433
Chronic Promotion*TP	-.116	.796	-.15	.885
Model for RQ1 (Promotion)				
Buying Impulsiveness	.055	.239	.23	.820
Chronic Prevention	-.543	.408	-1.33	.187
Time Pressure	-.049	.483	-.10	.919
RF Condition	-1.176	.504	-2.33	.022
Chronic Promotion	1.649	.635	2.60	.011
TP*RF	.326	.696	.47	.640
Chronic Promotion*TP	-.289	1.110	-.26	.795
Chronic Promotion*RF	-1.940	1.098	-1.77	.080
Chronic Promotion*TP*RF	.889	1.573	.57	.573
Model for RQ1 (Prevention)				
Buying Impulsiveness	.056	.241	.23	.816
Chronic Promotion	.636	.398	1.60	.113
Time Pressure	-.066	.487	-.14	.893
RF Condition	-1.280	.513	-2.50	.014
Chronic Prevention	.710	.702	1.01	.314
TP*RF	.370	.700	.53	.599
Chronic Prevention*TP	-1.689	1.150	-1.47	.145
Chronic Prevention*RF	-1.213	1.101	-1.10	.273
Chronic Prevention*TP*RF	1.347	1.608	.84	.404

Table MDA6

Unstandardized regression coefficients and statistics for models calculated in Experiment 2.

	B	*SE*	*t*	*p*
Model for H2 (Promotion)				
Buying Impulsiveness	.083	.187	.44	.660
Cognitive Load	-.279	.190	-.147	.145
Chronic Prevention	.209	.170	1.23	.221
Chronic Promotion	.045	.259	.17	.863
Chronic Promotion*CL	-.165	.371	-.44	.657
Model for H2 (Prevention)				
Buying Impulsiveness	.082	.187	.44	.661
Cognitive Load	-.279	.190	-.147	.144
Chronic Promotion	-.018	.190	-.09	.925
Chronic Prevention	.034	.243	.14	.888
Chronic Prevention*CL	.351	.336	1.04	.299
Model for RQ1 (Promotion)				
Buying Impulsiveness	.087	.193	.45	.652
Chronic Prevention	.217	.174	1.25	.214
Cognitive Load	-.286	.271	-1.06	.293
RF Condition	.087	.278	.31	.755
Chronic Promotion	.228	.408	.56	.576
CL*RF	.002	.388	.01	.995
Chronic Promotion*CL	-.131	.608	-.22	.830
Chronic Promotion*RF	-.313	.533	-.59	.559
Chronic Promotion*CL*RF	-.036	.778	-.05	.963
Model for RQ1 (Prevention)				
Buying Impulsiveness	.114	.187	.61	.542
Chronic Promotion	.012	.189	.06	.951
Cognitive Load	-.296	.265	-1.11	.267
RF Condition	.101	.271	.37	.709
Chronic Prevention	.499	.307	1.62	.107
CL*RF	-.028	.380	-.07	.940
Chronic Prevention*CL	-.169	.431	-.39	.696
Chronic Prevention*RF	-1.227	.498	-2.46	.015
Chronic Prevention*CL*RF	1.364	.686	1.99	.049

Table MDA7

Unstandardized regression coefficients and statistics for models calculated in Mega-Analysis.

	B	*SE*	*t*	*p*
Model for RQ1 (Promotion)				
Buying Impulsiveness	.025	.019	1.28	.202
Chronic Prevention	.019	.030	.63	.531
Resource Constraint	-.026	.043	-.61	.545
RF Condition	-.064	.044	-1.45	.147
Chronic Promotion	.121	.059	2.04	.043
RC*RF	.020	.062	.33	.741
Chronic Promotion*RC	-.034	.095	-.35	.725
Chronic Promotion*RF	-.163	.085	-1.91	.057
Chronic Promotion*RC*RF	.053	.127	.42	.677
Model for RQ1 (Prevention)				
Buying Impulsiveness	.018	.019	.96	.338
Chronic Promotion	.029	.031	.94	.349
Resource Constraint	-.032	.043	-.75	.453
RF Condition	-.062	.044	-1.40	.162
Chronic Prevention	.117	.053	2.20	.028
RC*RF	.018	.061	.29	.769
Chronic Prevention*RC	-.077	.077	-.99	.322
Chronic Prevention*RF	-.259	.084	-3.10	.002
Chronic Prevention*RC*RF	.237	.117	2.03	.044
